# Protein Digestion Kinetics Influence Maternal Protein Loss, Litter Growth, and Nitrogen Utilization in Lactating Sows

**DOI:** 10.3389/fnut.2022.862823

**Published:** 2022-03-21

**Authors:** Hao Ye, Pieter Langendijk, Neil W. Jaworski, Yujun Wu, Yu Bai, Dongdong Lu, Greg Page, Bas Kemp, Dandan Han, Nicoline M. Soede, Junjun Wang

**Affiliations:** ^1^State Key Laboratory of Animal Nutrition, China Agricultural University, Beijing, China; ^2^Adaptation Physiology, Wageningen University and Research, Wageningen, Netherlands; ^3^Trouw Nutrition R&D, Amersfoort, Netherlands

**Keywords:** lactating sow, weight loss, litter performance, protein digestion kinetics, protein utilization

## Abstract

Body protein losses in lactating sows have a negative impact on sow and litter performance. Improving dietary amino acid utilization may limit protein mobilization. The effects of dietary protein kinetics on sow body condition loss, blood plasma metabolites, and plasma insulin-like growth factor-1 (IGF-1), and also on litter gain during lactation, were investigated in this study. In total, 57 multiparous sows were fed one of three lactation diets with the same crude protein level: low level of slow protein diet (LSP) (8% slowly degradable protein of total protein), medium level of slow protein diet (MSP) (12% slowly degradable protein of total protein), or high level of slow protein diet (HSP) (16% slowly degradable protein of total protein) in a complete block design. Our results showed that HSP sows lost the least body weight compared to MSP and LSP sows (11.9 vs. 17.3 and 13.5 kg, respectively; *p* = 0.01), less body protein than MSP sows (1.0 vs. 2.1 kg; *p* = 0.01), and tended to lose less loin muscle thickness than LSP sows (1.7 vs. 4.9 mm; *p* = 0.09) between Day 2 to Day 21 post-farrowing. LSP sows had greatest plasma urea level on Day 6 than MSP and HSP sows (4.9 vs. 3.6 and 3.1 mmol/L, respectively; *p* < 0.01) and on Day 13 (5.6 vs. 4.1 and 3.7 mmol/L, respectively; *p* < 0.01). HSP sows had the lowest plasma urea level at Day 20 compared to LSP and MSP sows (4.0 vs. 5.5 and 4.9 mmol/L, respectively; *p* < 0.01). The average plasma urea level of Days 6, 13, and 20 post-farrowing was negatively correlated with slow protein intake (*r* = −0.49, *p* < 0.01). Litter gain, milk composition, and nitrogen output to the environment did not differ significantly among the treatment groups. Therefore, the dietary protein kinetics affected mobilization of maternal reserves in multiparous sows during lactation, with a high fraction of slow protein-sparing protein mobilization.

## Introduction

During lactation, sows mobilize their body reserves to support milk production as the voluntary feed intake does not cover their nutritional demands during this period (1). As litter size has continuously increased with genetic selection during the last decades (2), the heavier lactational burden increases concerns over higher body loss of modern hybrid sows in lactation. Sow body loss in lactation has been associated with negative impacts on post-lactational reproductive metrics, which include prolonged weaning-to-estrus interval, anestrus, reduced farrowing rate, and reduced litter size in the subsequent cycle (2–4). It has been indicated that high body protein tissue mobilization in modern sows is associated with impaired follicular development and reduced milk production (4–6). Increasing dietary protein levels can be effective in saving sow body protein mobilization and increasing milk production during lactation (3, 7, 8), but may bring negative impacts on the environment, as the nitrogen output in animal excreta has been recognized as a threat to the quality of water, air, and soil (9). In this respect, improvement in dietary protein utilization efficiency is needed to balance production efficiency and nitrogen output to the environment to ensure sustainable swine production.

The proteolysis pattern of proteins in the small intestine can vary greatly among different protein sources, and these differences can affect protein utilization. For example, whey and soy proteins are rapidly digested, whereas in comparison, casein and milk proteins are digested more slowly by humans (10, 11). Studies that investigate protein gastrointestinal kinetics in humans (10–13) indicated that proteins with a slower digestion rate may save body protein from breakdown, but the effect was dependent on the physiological status of subjects, for example, age. These studies focused only on short-term effects in the postprandial period, and information on such effects in lactating sows is lacking. We therefore performed a meta-analysis on data derived from 21 studies in lactating sows from the last 20 years with varying dietary protein sources. Protein sources used in these manuscripts were evaluated according to the fractions of slow and fast protein, based on *in vitro* protein degradation characteristics of a range of ingredients previously established by a modified method based on (14). In this meta-analysis, a higher ratio of slow protein to total dietary protein (8–16%) appeared to reduce sow body protein loss during lactation, which is in agreement with the previous studies in humans (11, 12).

In this study, the effect of protein digestion kinetics on sow and litter performance was investigated by feeding the lactating sows with diets in different ratios of slow protein to total protein, over the range of ratios found in our meta-analyses: 8, 12, and 16%. Sow body weight loss, milk production, blood metabolites, litter gain, and nitrogen loss to the environment during lactation were evaluated. We hypothesized that sows fed diets with increased levels of slow protein would experience reduced body protein mobilization during lactation and increased nitrogen efficiency.

## Materials and Methods

### Animals, Housing, and Management

Experiments were conducted at the swine research unit of China Agricultural University (Chengde, China). The animal use protocols were reviewed and approved by the China Agricultural University Animal Care and Use Committee (Beijing, China).

A total of 57 large White × Landrace sows that had been inseminated with Duroc semen were used in this study. During gestation, sows were housed in individual stalls with room temperature at 20°C. The sows were fed a commercial gestating diet [NRC (15)] at 2.0 kg/day during the early gestation (days 0–85) and 3.0 kg/day during late gestation (days 86–107), in equal portions three times a day, at 5:30, 10:30, and 16:30.

Sows were moved to the farrowing rooms at 8.4 ± 3.8 days prior to the expected farrowing date. The room temperature of the farrowing rooms was strictly controlled at 20°C and lights were on from 6:00 to 16:00.

After parturition (Day 0), piglets were treated according to the routine management on the farm, which included teeth clipping, tail docking, ear notching, and subcutaneous iron dextran injections (200 mg/pig), a routine procedure to prevent iron deficiency within 24 h after birth. For sows that completed parturition after 16:30, the next day was considered as Day 0. On Day 2 post-farrowing, an attempt was made to standardize the litter size of sows to 13 piglets [litter size = 13 (40 litter), 14 (16 litter), and 15 (1 litter)]. Piglets received creep feed from Day 7 onward.

Once all sows within a farrowing room had reached Day 21 post-farrowing, all sows in the farrowing room were weaned, at 24.5 ± 2.5 days post-farrowing.

### Feeding and Diets

Based on the equal distribution of parity (4.6 ± 0.7) and body weight on the day of entering the farrowing crate (263.4 ± 2.9 kg), sows were allocated to one of three dietary treatments; low (LSP, *n* = 19), mid (MSP, *n* = 19), or high (HSP, *n* = 19) levels of slow protein, in which the level of slow protein to total dietary protein was 8, 12, and 16%, respectively, based on the range of ratios found in the meta-analysis, in a complete block design. The LSP and HSP diets were formulated as shown in Table 1 and mixed in equal amounts to feed sows in the MSP group. All diets were formulated to contain equal quantities of net energy, standardized ileal digestible amino acid, and apparent total tract digestible phosphorus.

**TABLE 1:** Ingredients and nutrient composition of experimental diets with 8% slow protein (LSP) and 16% slow protein (HSP) of total protein.

Ingredient, %	LSP	HSP
Wheat	58.9	23.1
Wheat bran	20.2	21.9
Sunflower meal 35% CP	7.20	–
Rapeseed meal < 38% CP	–	9.06
Maize	3.25	36.4
Soybean oil	2.90	2.07
Soy protein concentrate	–	3.17
Maize Gluten 60%	2.49	–
Calcium carbonate fine	1.34	–
Premix	1.00	1.00
Monocalcium phosphate (fine)	0.986	0.922
Calcium carbonate fine	–	1.26
Salt (Nacl) fine	–	0.243
Soybean meal 43, 50 < Cfiber < 70	0.659	–
AA L-Lysine HCl 98%	0.541	0.369
Na bicarbonate	0.311	0.325
L -Threonine 98%	0.158	0.117
AA L-Isoleucine 98.5%	0.037	0.028
L-Valine 96.5%	0.037	0.014
AA L-Tryptophan 98%	0.009	0.015
AA DL-Methionine 99%	–	0.007
Total	100	100
**Composition (calculated)**		
DM, %	88.3	88.3
CP, %	15.0	15.0
NE, kCal/kg	2.25	2.25
TDF, %	19.8	19.8
SID LYS, g/kg	7.74	7.74
SID M + C, g/kg	4.64	4.64
SID THR, g/kg	5.18	5.18
SID TRP, g/kg	1.47	1.47
SID VAL, g/kg	5.65	5.65
SID ILE, g/kg	4.64	4.64
SID LEU, g/kg	9.29	9.62
Slow Protein, % of total protein	8	16
**Composition (analyzed)**		
CP, %	17.5	16.8
ME, MJ/kg	13.8	13.1

After entering the farrowing crates, sows received 1 kg/day of the MSP diet to adapt to the dietary ingredients before parturition. On Day 1 post-farrowing, sows received 2.0 kg/day of one of the experimental lactation diets according to the treatment, and daily feed allowance was increased by 0.5 kg/day. For the first 16 sows entering into the study, the daily feed allowance was given according to the litter size carried. Sows with 14 or more piglets were fed up to 8 kg/day, and sows with 13 piglets were fed up to 7.5 kg/day. Sows having 12 or less piglets were fed up to 6.5 kg/day, and the feed allowances were adjusted daily to the litter size if piglets dropped out of the litters. However, as 3 of the first 16 sows appeared to be gaining weight, the feed allowance for the remaining sows was 2.0 kg on Day 2 and gradually increased with steps of 0.5–6.0 kg/day on Day 9 post-farrowing. After Day 9, feeding levels were maintained until weaning. Sows had free access to water throughout the experimental period. Feed refusals were removed from the trough before feeding in the morning and weighed. The daily feed intake was calculated as feed allowance minus feed refusal. If feed refusals were wet, a 300–400 g representative sample was taken and placed in a drying oven for 72 consecutive hour at 65°C and another 24 h at 25°C, to establish dry weight and calculate feed refusals this way.

### Body Weight, Back Fat, and Loin Muscle

The body weight, backfat thickness, and loin muscle thickness of sows and body weight of individual piglets were measured on Days 2, 7, 14, and 21 post-farrowing before the last meal of the day. The thicknesses of sow backfat and loin muscle were measured with a type-B ultrasonic device (MyLab™ touchVet, Esaote S.p.A, Genoa, Italy) equipped with a 15-cm, 3.5 Hz probe. The probe was placed 50 mm from the midline at the last rib and the backfat and loin muscle thicknesses were recorded at both sides of the sow.

### Collection of Blood, Milk, and Feces

On Days 6, 13, and 20 post-farrowing, a total of 5 ml of blood was collected from each sow *via* an ear marginal vein 4 h after the first morning feeding. Filled tubes were placed in an ice box for transportation to the laboratory and centrifuged at 3,000 × g for 15 min at 4°C. After centrifugation, plasma samples were equally split into two storage tubes and then stored at −80°C prior to the non-esterified fatty acid (NEFA), urea, creatinine (CREA), and insulin-like growth factor-1 (IGF-1) analyses.

On Days 6, 13, and 20 post-farrowing, a total of 40 ml of milk was collected from each sow. First, 2 ml of oxytocin (20–40 IU) was injected through the ear marginal vein to induce milk letdown, and then, milk was obtained in equal proportions from all functional teats and pooled in 50-ml tubes for each sow. Samples were divided into two portions with equal volume and stored at −20°C until the analysis of milk lactose, fat, and protein.

On Days 6, 13, and 20 post-farrowing, 200–400 g fresh fecal samples was collected in aseptic bags for each sow and stored at –20°C. Before analyses, all fecal samples were thawed and placed in a drying oven for 72 consecutive hour at 65°C and another 24 h at 25°C.

### Chemical Analyses

The Kjeldahl method [Method 984.13, AOAC (16), Foss™ Kjeltec™ 8100 Manual Distillation Unit, Foss Analytical A/S, Hillerød, Denmark] was used to determine the nitrogen concentration of feed and fecal samples, and protein was calculated as N × 6.25. Gross energy levels of feed and fecal samples were analyzed with an Automatic Isoperibol Oxygen Bomb Calorimeter (Parr 6400 Calorimeter, Moline, IL, United States) The levels of acid-insoluble ashes (AIA) of feed and fecal samples were measured as described previously (17), to be used as an internal marker determining protein and energy digestibility.

Sow blood plasma samples were thawed before analyses. The NEFA, urea, and CREA levels in sow blood plasma were measured using corresponding commercial colorimetric kits (NEFA: Nanjing Jiancheng Bioengineering Institute, Nanjing, China; Urea and CREA: Beijing Labbang Technologies Corporation, Beijing, China) with an automatic biochemical analyzer (Hitachi 7160, Hitachi High-Technologies Corporation, Tokyo, Japan). The level of IGF-1 in sow plasma was detected by Iodine [125I] Insulin Radioimmunoassay Kit (Tianjin Jiuding Medicine Biotechnology Corporation, Tianjin, China) with a gamma radioimmunoassay counter (GC-2010, Anhui USTC Zonkia Scientific Instruments Corporation, Anhui, China).

Sow milk samples were thawed and thoroughly mixed before analyses. Milk protein, fat, and lactose levels were determined by an online somatic cell count tester (Combi-System with SomaCount FC and DairySpec FT, Bentley Instruments, Inc., Chaska, MN, United States).

### Calculations

The digestibility of protein and energy was calculated as follows:

Digestibility (%) =  100-[(CIinput×CCoutput)/(CCinput×CIoutput)× 100], where CIinput and CIoutput were the concentration of AIA in feed and feces, respectively; CCinput and CCoutput were the concentration of components in feed and feces, respectively.

The metabolizable energy (ME) intake of a sow was calculated as follows: (gross energy of diets, MJ/kg × energy digestibility of corresponding diet, % × 0.999) – (0.82 × crude protein of diets, g/kg × corresponding protein digestibility × 4.184) × average feed intake, kg/day (18).

Body protein and fat reserves of sows were estimated as follows: protein (kg) = 2.28 + (0.178 × 0.96 × SBW, kg) − (0.333 × BF, mm) and fat (kg) = −26.4 + (0.221 × 0.96 × SBW, kg) + (1.331 × BF, mm), where SBW represents sow body weight and BF represents sow back fat (19).

The litter gain was calculated as the sum of the individual piglet gains for separate periods between Days 2 and 21. If piglet gain was negative due to decreased body weight in a certain lactating period, it was corrected to 0 in the calculation, and the body weight of piglets taken out of the experiment because of death or weakness was recorded for the gain until removal.

The total nitrogen loss of sows to the environments (g) was calculated as dietary nitrogen intake, g+ nitrogen mobilized from body reserves, g– nitrogen output in milk, g, where nitrogen mobilized from body reserves = estimated protein loss/6.25, and the total nitrogen output in milk was calculated as follows: milk nitrogen output = [(0.0257 × AGD, g/d) + (0.42 + LS)] × 19, day (20). The nitrogen output in feces (g) was calculated as total dietary nitrogen intake, g × (1 – dietary protein digestibility), and the nitrogen in urine as total nitrogen loss, g – nitrogen in feces, g.

Additionally, the protein efficiency was defined as follows: protein output in milk, g/d/(protein intake, g/d+ protein loss, g/d– protein for sow maintenance, g/d, where protein for sow maintenance = 1.32 × [(SBW2 + SBW21)/2] 0.75 (21).

### Statistics

Relationships between sow and litter characteristics were analyzed with SAS OnDemand for Academics (SAS Institute Inc., Cary, NC, United States), using generalized linear model (GLM) and mixed models. Model assumptions were checked using the univariate procedure.

To test the effects of slow protein level in the diet and parity classes on sow body loss between Days 2 and 21 post-farrowing, the following GLM model was used:


Y=μ+treatment+parity⁢class+parity⁢class

*treatment+LS21+SBW2+e⁢(model⁢ 1)

where Y = sow body weight/estimated protein/estimated fat loss from Days 2 to 21 post-farrowing, μ = overall mean, treatment = diet with low/mid/high level of slow protein [LSP (*n* = 19), MSP (*n* = 19), HSP (*n* = 19)], parity class = parity class [3 + 4 (*n* = 16) and 5 (*n* = 42)], LS21 = litter size on Day 21 post-farrowing, SBW2 = sow body weight on Day 2 post-farrowing, e = residual error. Sows that gain weight between Days 2 and 21 post-farrowing were not included in the model.

Subsequently, a series of models were established based on the model 1. When comparing feed, ME, and digestible protein intake of sows in different dietary treatments, LS21 and SBW2 were taken out of model 1.

For effects of slow protein level in diet and parity class on sow backfat and loin muscle levels between Days 2 and Day 21 post-farrowing, model 1 was used and SBW2 was replaced by BF2 (sow backfat level on Day 2 post-farrowing) and LM2 (sow loin muscle level on Day 2 post-farrowing), respectively.

For effects of slow protein level in diet and parity classes on litter weight gain between Days 2 and 21, model 1 was used and SBW2 was replaced by LW2 (litter weight Day 2), and LS21 was taken out of the model. Same model was used to compare the litter weight gain in the different weeks.

To test the effects of level of slow protein in diet and parity class on sow blood plasma IGF-1, NEFA, CREA, and urea levels, the milk composition that includes milk fat, protein and lactose levels on Days 6, 13, and 20 post-farrowing, the nitrogen loss to environments, nitrogen output in feces or urine, and the maternal protein efficiency, both the SBW2 and LS21 were excluded from model 1.

For all models, the interaction between treatment and parity class was excluded from the model if it was not significant. Results are presented as least square means ± SEM in all GLM models.

Besides, the relationships among sow plasma IGF-1, urea, CREA, NEFA, and sow body loss were tested in mixed models as follows:


Y=μ+Bodyweightloss/LMloss/BFloss/

estimated⁢protein⁢loss/estimated⁢fat⁢loss+week+e

where Y = plasma IGF-1/urea/CREA/NEFA, the lactating stages [i.e., week 1 (= Day 6)/2 (= Day 13)/3 (=Day 20)] were described as repeated unit in the model statements. Results are presented as least square means ± SEM in all mixed models.

The Pearson’s correlation tests were performed to test the correlations between the mean level of plasma urea/CREA/NEFA/IGF-1 from Days 6 to 20 and the sow protein intake (total/slow/fast), weight loss, loin muscle loss, backfat loss, estimated protein, and fat loss between Days 2 and 21.

## Results

### Feed Intake

The 57 sows completed the experimental period with an average feed intake of 5.17 ± 0.04 kg/day between Days 2 and 21 post-farrowing. In weeks 1, 2, and 3 post-farrowing, average sow feed intake was 3.22 ± 0.01, 5.74 ± 0.05, and 6.27 ± 0.09 kg/day, respectively. No differences were found in sow feed, digestible protein, and ME intake between different diets (Table 2).

**TABLE 2:** Feed, metabolizable energy (ME), and digestible protein intake between Days 2 and 21 post-farrowing in sows fed with different slow protein levels (LSmeans ± SEM).

	Diets in lactation				*P*-Value	
	LSP	MSP	HSP	SEM	*D*	*P*
Feed intake, kg/d	5.1	5.1	5.3	0.1	0.17	0.26
ME intake, MJ/d^a^	70.5	69.2	69.6	1.0	0.64	0.25
Digestible protein intake, g/d^b^	797.5	778.4	778.6	11.3	0.40	0.24

*D, diet [LSP/MSP/HSP, where LSP, low level of slow protein diet (8%); MSP, medium level of slow protein diet (12%); HSP, high level of slow protein diet (16%); P, parity class (4/5, where 4 = sows in parity 3 and parity 4; 5 = sows in parity 5)]. ^a^Calculations based on apparent energy digestibility LSP/MSP/HSP = 84.8/83.4/83.0%, respectively. ^b^Calculations based on apparent protein digestibility LSP/MSP/HSP = 89.1/87.8/87.3%, respectively.*

### Sow Body Weight, Backfat, and Loin Muscle Loss

The body weight loss, loin muscle loss, and estimated protein loss between Days 2 and 21 post-farrowing were lowest or tended to be lowest in sows fed with HSP. HSP sows lost significantly less body weight than MSP sows (Table 3; Δ = 5.1 kg, *p* = 0.01) and tended to lose less loin muscle thickness than sows fed with LSP (Table 3; Δ = 2.2 mm, *p* = 0.09). Estimated protein losses were highest in sows fed with MSP (Table 3). Estimated fat loss and backfat loss, calculated protein and fat loss, and protein efficiency were not different among the dietary treatments (Table 3).

**TABLE 3:** Effects of slow protein level in the lactating sow diet on sow body condition parameters on Days 2, 21, and 2–21 post-farrowing (LSmeans ± SEM).

	Diets in lactation				*P*-Value	
	LSP	MSP	HSP	SEM	*D*	*P*
Sows (*n*)	17	18	19			
**Day 2 post-farrowing^c^**						
Body weight, kg	260.4	265.4	264.4	5.1	0.76	0.49
LM, mm	54.3	56.4	56.4	1.1	0.28	0.24
BF, mm	17	18.4	17.7	1.2	0.74	0.81
Protein, kg	41.1	41.5	41.6	0.7	0.87	0.31
Fat, kg	51.5	54.4	53.2	2.4	0.71	0.88
**Day 21 post-farrowing^c^**						
Body weight, kg	249.2	248.2	251.9	4.9	0.86	0.25
LM, mm	50.7	53.2	54.9	1.2	0.05	0.64
BF, mm	14.4	15.6	14.3	0.9	0.54	0.87
Protein, kg	40.1	39.5	40.6	0.7	0.59	0.26
Fat, kg	45.6	47	46.1	2.2	0.88	0.38
**Day 2 to Day 21**						
Body weight loss, kg	13.5^ab^	17.3^a^	11.9^b^	1.6	0.04	0.29
LM loss, mm	4.9^x^	3.4^xy^	1.7^y^	1.0	0.09	0.49
BF loss, mm	2.9	2.4	3.1	0.4	0.50	0.13
Estimated protein loss, kg	1.4^a^	2.1^b^	1.0^a^	0.3	0.01	0.94
Estimated fat loss, kg	6.5	7.0	6.4	0.9	0.87	0.08
Protein efficiency, %^c^	63.2	66.1	68.4	2.6	0.38	0.73

*D, diet [LSP/MSP/HSP, where LSP = low level of slow protein diet (8%); MSP, medium level of slow protein diet (12%); HSP, high level of slow protein diet (16%); P, parity class (4/5, where 4 = sows in parity 3 and parity 4; 5 = sows in parity 5)]; LM, loin muscle thickness; BF, backfat thickness. ^a,b^LSmeans within a row with different superscripts differ significantly at p < 0.05. ^x,y^LSmeans within a row with different superscripts differ significantly at p < 0.1. ^c^LSmeans compared in models with treatments and sow parity class as explaining variables.*

As litter size was included as covariate in model 1, every extra-piglet on Day 21 was estimated to result in 3.0 kg more sow body weight loss (*p* < 0.01), 0.5 kg more sow body protein loss (*p* < 0.01), and 0.8 kg more sow body fat loss (*p* < 0.01) between Days 2 and 21, respectively. Moreover, sows with higher backfat or loin muscle thicknesses on Day 2 were estimated to lose more backfat (β = 0.3 mm/mm; *p* < 0.01) or loin muscle (β = 0.3 mm/mm; *p* < 0.01) between Days 2 and 21 of lactation.

### Litter Growth

Mean litter gain from Days 2 to 21 was 54.3 ± 1.2 kg and was 12.5 ± 0.4 kg in week 1 (Days 2–7), 21.4 ± 0.7 kg in week 2 (Days 7–14), and 20.3 ± 0.5 kg in week 3 (Days 14–21). The litter growth of sows fed different diets did not differ significantly between Days 2 and 21 (Table 4). However, in week 2 post-farrowing, MSP litters tended to have a greater gain than litters of sows fed with LSP (Δ = 2.8 kg, *p* = 0.06). The parity of sows did not have significant effects on litter growth performance either between Days 2 and 21 or different weeks.

**TABLE 4:** Effects of slow protein level in the lactation diet and parity on litter gain between Days 2 and 21 post-farrowing, in weeks 1, 2, and 3 post-farrowing (LSmeans ± SEM).

	Diet in lactation			*p*-Value	
	LSP	MSP	HSP	SEM	*D*	*P*
Litters (*n*)	19	19	19			
**Day 2 post-farrowing^c^**						
Litter size	13.1^x^	13.5^y^	13.3^xy^	0.1	0.09	0.92
Litter weight, kg	22.8	23.0	22.8	0.7	0.95	0.23
Day 21 post-farrowing^c^						
Litter size	12.0	12.4	12.2	0.3	0.70	0.44
Litter weight, kg	72.8	78.8	74.8	2.7	0.26	0.87
**Day 2 to Day 21,**						
Litter gain week 1, kg	12.3	12.8	12.4	0.6	0.80	0.30
Litter gain week 2, kg	20.2^x^	23.0^y^	21.3^xy^	0.8	0.06	0.40
Litter gain week 3, kg	19.0	21.2	20.0	0.8	0.23	0.43
Litter gain (day 2–21), kg	52.2	56.4	53.5	1.6	0.18	0.19

*D, diet [LSP/MSP/HSP, where LSP, low level of slow protein diet (8%); MSP, medium level of slow protein diet (12%); HSP, high level of slow protein diet (16%); P, parity class (4/5, where 4 = sows in parity 3 and parity 4; 5 = sows in parity 5)]. ^x,y^LSmeans within a row with different superscripts differ significantly at p < 0.1. ^c^LSmeans compared in models with treatments and sow parity class as explaining variables.*

### Milk Composition

On Day 6 post-farrowing, sows fed with MSP tended to have a higher milk fat concentration compared to the sows fed with LSP (Δ = 1.6%, *p* = 0.09; Table 5). On Days 13 and 20, no significant differences in terms of the milk composition were found.

**TABLE 5:** Sow milk fat, protein, and lactose concentrations on Days 6, 13, and 20 post-farrowing (LSmeans ± SEM).

	Diet in lactation				*p*-Value	
	LSP	MSP	HSP	SEM	*D*	*P*
**Day 6 post-farrowing^c^**						
Sows (*n*)	15	18	16			
Milk fat,%	5.6^x^	6.9^y^	5.8^xy^	0.7	0.09	0.45
Milk protein,%	4.1	4.5	4.0	0.3	0.21	0.21
Milk lactose,%^d^	5.6	5.5	5.2	0.3	0.50	<0.01
**Day 13 post-farrowing^c^**						
Sows (*n*)	17	18	15			
Milk fat,%	4.9	5.2	5.3	0.4	0.80	0.71
Milk protein,%	3.5	3.8	3.7	0.2	0.62	0.74
Milk lactose,%	5.6	6.0	5.8	0.3	0.62	0.24
**Day 20 post-farrowing^c^**						
Sows (*n*)	16	17	15			
Milk fat,%	5.0	5.4	4.9	0.5	0.70	0.37
Milk protein,%	3.6	4.0	3.8	0.3	0.68	0.42
Milk lactose,%	6.0	6.1	5.9	0.3	0.95	0.78

*D, diet [LSP/MSP/HSP, where LSP, low level of slow protein diet (8%); MSP, medium level of slow protein diet (12%); HSP, high level of slow protein diet (16%); P, parity class (4/5, where 4 = sows in parity 3 and parity 4; 5 = sows in parity 5)]. ^x,y^LSmeans within a row with different superscripts differ significantly at p < 0.1. ^c^LSmeans compared in models with treatments and sow parity class as explaining variables. ^d^Milk lactose level of sows in parity class 4 was lower than parity class 5 (4.9 < 6.0%, p < 0.01).*

### Plasma Metabolites and Insulin-Like Growth Factor-1

On Day 6, sows fed LSP had a higher urea level in plasma than sows fed MSP and HSP (Δ = 1.3, *p* < 0.01, Δ = 1.8, *p* < 0.01, respectively; Table 6), but had a lower level of NEFA compared to the sows fed with HSP (Δ = 0.1, *p* = 0.03; Table 6). On Day 13, sows fed with LSP had higher level of urea than sows in MSP and HSP group (Δ = 1.5, *p* < 0.01, Δ = 1.9, *p* < 0.01, respectively; Table 6). On Day 20, both LSP and MSP sows had higher plasma urea level than HSP sows (Δ = 1.5, *p* < 0.01, Δ = 0.9, *p* = 0.05, respectively, Table 6). The plasma CREA and IGF-1 levels were not different. The parity of sows did not have significant effects on blood parameters on Days 6, 13, and 20, except sows in parity class 4 tended to have higher IGF-1 level than sows in parity class 5 on Day 20.

**TABLE 6:** Sow plasma IGF-1 (ng/mL), urea (mmol/L), CREA (μmol/L), and NEFAs (mmol/L) concentrations on Days 6, 13, and 20 post-farrowing (LSmeans ± SEM).

	Diets in lactation				*p*-Value	
	LSP	MSP	HSP	SEM	*D*	*P*
**Day 6 post-farrowing^c^**						
Sows (*n*)	18	18	16			
IGF-1, ng/mL	90.2	93.1	81.5	6.1	0.39	0.68
Urea, mmol/L	4.9^a^	3.6^b^	3.1^b^	0.3	<0.01	0.37
CREA, μmol/L	169	172	160	6.2	0.39	0.66
NEFA, μmol/L	251^x^	323^xy^	408^y^	47.9	0.08	0.43
**Day 13 post-farrowing^c^**						
Sows (*n*)	17	18	15			
IGF-1, ng/mL	84.3	77.1	75.2	4.2	0.28	0.75
Urea, mmol/L	5.6^a^	4.1^b^	3.7^b^	0.3	<0.01	0.49
CREA, μmol/L	164	171	155	5.2	0.12	0.73
NEFA, μmol/L	212	273	272	37.6	0.41	0.84
**Day 20 post-farrowing^c^**						
Sows (*n*)	16	17	17			
IGF-1, ng/mL	73.4	70.7	76.4	4.3	0.63	0.07
Urea, mmol/L	5.5^a^	4.9^a^	4.0^b^	0.3	<0.01	0.28
CREA, μmol/L	172	173	164	6.3	0.52	0.81
NEFA, μmol/L	254	323	266	48.6	0.55	0.28

*D, diet [LSP/MSP/HSP, where LSP, low level of slow protein diet (8%); MSP, medium level of slow protein diet (12%); HSP, high level of slow protein diet (16%); P, parity class (4/5, where 4 = sows in parity 3 and parity 4; 5 = sows in parity 5)]. ^a,b^LS means within a row with different superscripts differ significantly at p < 0.05. ^x,y^LS means within a row with different superscripts differ significantly at p < 0.1. ^c^LSmeans compared in models with treatments and sow parity class as explaining variables. ^d^LSmeans compared in mixed models with week as explaining variable and repeated measures.*

### Nitrogen Loss to the Environments

Mean nitrogen loss to the environment between Days 2 and 21 post-farrowing was 998.8 ± 39.2 g and did not differ between the treatments (Table 7). However, sows fed with HSP had higher nitrogen output in feces than sows fed with MSP and LSP (Δ = 12 g, *p* < 0.01, Δ = 38 g, *p* < 0.01, Table 7). The estimated nitrogen output in urine did not differ significantly between treatments (Table 7).

**TABLE 7:** Total nitrogen loss, fecal nitrogen output, and urinary nitrogen output between Days 2 and 21 post-farrowing (LSmeans ± SEM).

	Diets in lactation				*p*-Value	
	LSP	MSP	HSP	SEM	*D*	*P*
Sows (*n*)	19	19	19			
Total nitrogen loss, g	1282	1256	1146	73	0.4	0.55
Fecal nitrogen, g	278^a^	304^b^	316^c^	4	<0.01	0.29
Urinary nitrogen, g	1003	953	832	71	0.24	0.58

*D, diet [LSP/MSP/HSP, where LSP, low level of slow protein diet (8%); MSP, medium level of slow protein diet (12%); HSP, high level of slow protein diet (16%); P, parity class (4/5, where 4 = sows in parity 3 and parity 4; 5 = sows in parity 5)]. ^a–c^LSmeans within a row with different superscripts differ significantly at p < 0.05.*

### Relationships Between Sow Plasma Parameters and Performance

Sow protein intake (total/slow/fast), plasma urea, CREA, NEFA, and IGF-1 levels as measured on Days 6, 13, and 20 were correlated with the loss of sow weight, loin muscle, backfat, estimated protein, and fat in mixed models, with the measurements in different weeks being considered as repeated observations (Table 8). Results showed that daily slow protein intake was negatively correlated with the plasma urea level (Table 8), whereas total protein intake was positively correlated with plasma urea. CREA level was positively correlated with the estimated protein loss (Table 8). NEFA level was negatively correlated with the total digestible protein intake, fast protein intake, but positively correlated with the slow protein intake, sow body weight loss, sow backfat loss, and sow estimated fat loss (Table 8). IGF-1 levels were not found to correlate with the sow protein intake and sow body loss.

**TABLE 8:** Relationships between sow blood plasma urea (mmol/L), CREA (μmol/L), NEFA (μmol/L), and IGF-1 (ng/ml) levels and sow total protein intake (g/d), sow slow protein intake (g/d), sow fast protein intake (g/d), sow body weight (kg), loin muscle (mm), backfat (mm), estimated body protein (kg), and estimated body fat (kg) loss in a 21-day lactating period.

Item	Urea			CREA			NEFA			IGF-1		
	*r* ^a^	β ^b^	P^b^	*r* ^a^	β ^b^	P^b^	*r* ^a^	β ^b^	P^b^	*r* ^a^	β ^b^	P^b^
Total digestible protein intake, g/d	0.34	0.004	0.02	0.16	0.002	0.61	–0.3	< −0.001	<0.01	0.08	0.05	0.63
Slow protein intake, g/d	–0.49	–0.01	<0.01	–0.1	–0.1	0.46	0.2	<0.001	0.1	–0.18	0.01	0.41
Fast protein intake, g/d	0.59	0.01	<0.001	0.2	0.02	0.54	–0.37	<0.001	<0.01	0.18	0.05	0.23
Sow body weight loss, kg	–0.09	–0.6	0.62	0.22	0.02	0.08	0.52	5.62	0.04	–0.32	–0.02	0.51
Sow loin muscle loss, mm	–0.12	–0.3	0.23	0.08	–0.01	0.11	0.16	1.39	0.09	0.06	–0.01	0.28
Sow backfat loss, mm	–0.38	–0.29	<0.01	0.03	0.003	0.67	0.43	2.71	<0.01	–0.12	–0.01	0.39
Sow estimated protein loss, kg	0.1	–0.05	0.26	0.22	0.004	0.02	0.32	0.56	0.46	–0.28	–0.01	0.68
Sow estimated fat loss, kg	–0.32	–0.52	0.01	0.12	0.01	0.79	0.55	4.68	<0.01	–0.23	–0.01	0.47

*CREA, creatinine; NEFA, non-esterified fatty acid; IGF-1, insulin-like growth factor-1. ^a^Pearson’s correlation coefficients between items and mean plasma urea, CREA, NEFA, and IGF-1 levels over Days 6, 13, and 20. ^b^Estimate and p-value of mixed models as: Urea/CREA/NEFA/IGF-1 = items + week, with week as repeated measures.*

In separate weeks, simple correlation tests showed that urea levels on Days 6, 13, and 20 were positively correlated with the fast protein intake in weeks 1, 2, and 3, respectively (*r* = 0.61, *p* < 0.01, *r* = 0.44, *p* < 0.01, *r* = 0.40, *p* < 0.01, in weeks 1, 2, and 3, respectively), but decreased with the increasing level of slow protein intake (*r* = −0.58, *p* < 0.01, *r* = −0.60, *p* < 0.01, *r* = −0.49, *p* < 0.01, in weeks 1, 2, and 3, respectively). IGF1 was not found to correlate with either protein intake or sow body weight loss in all three separate weeks.

## Discussion

The aim of this study is to investigate how dynamics of protein digestion can affect protein utilization for milk production and litter gain, thereby affecting maternal protein loss. In agreement with our hypothesis, a high concentration of slow protein in the diet (16% of total protein) appeared to result in the lowest maternal body weight loss, lowest maternal protein mobilization, and lowest loin muscle loss during lactation. Also, other indicators that are related to dietary protein utilization, such as blood urea, and calculated nitrogen efficiency pointed toward a more efficient use of protein and sparing of maternal protein.

The effects of a high concentration of slow protein in the diets on body protein breakdown were confirmed in the previous research in humans. It had been showed that the ingestion of casein, a slow protein source, reduced body protein breakdown by 34% compared to whey protein, a fast protein source (12). Feeding casein to young men resulted in greater positive postprandial leucine balance over 7 h than feeding free amino acids with a faster digestion rate but identical amino acid composition to casein (22). Moreover, ingestion of whey protein in repeated meals induced a slower amino acid release pattern and inhibition of protein breakdown than the same amount of whey protein in one meal (22). Beneficial effects of slow protein digestion in terms of sparing body protein mass may be attributed to a sustained amino acid delivery to the peripheral circulation, as slow protein uptake (casein, whey protein in repeated meals) resulted in a moderate and prolonged plasma hyperaminoacidemia compared to fast protein (free amino acid, whey protein in one meal) (22). Conversely, fast protein ingestion would induce a strong and transient amino acid delivery after ingestion (23), whereas only a part of these amino acids could be deposited as protein since intracellular amino acid concentration outreached the maximum for processing protein synthesis (24), and the surplus amino acids would be catabolized in the splanchnic bed as energy source. Soy protein, which can be rapidly digested and absorbed, would preferably be locally catabolized (up to 30%) after uptake by human, which results in higher urea production, and limit the peripheral accretion of soybean proteins (down to –20%) compared to milk protein which has a slower digestion rate (11). This finding was also supported by the increase in urea production with increasing level of fast protein intake in this study. Slow protein intake could induce sustained amino acid delivery to periphery, which improves the protein synthesis, which leads to inhibition of body protein break down.

In our study, the total weight loss of sows during lactation did not follow a linear response to increasing level of slow protein, as the weight loss of sows fed a low, medium, and high levels of slow protein was 13.5, 17.3, and 11.9 kg, respectively. The litters of sows fed the low level of slow protein in this study gained 8% less than litters of sows fed the medium level of slow protein, and those sows fed the low level of slow protein had 2% greater digestible protein intake, due to differences in digestibility of slow and fast protein diets, which might lead to less lactational burden and higher total amino acid availability for these sows, sparing body reserves mobilization. However, when including litter gain and/or protein intake into the model that compared sow weight loss between treatments, these two factors were not significant, and the least square means of sow weight loss did not turn to a linear response to slow protein level in the diet.

In this study, parity did not show any effects on sow body losses, protein utilization efficiency, and any interaction with the impacts of the level of slow protein in the sow diet. Previous studies on humans stated that the effects of slow and fast protein seen in younger subjects might be weakened with aging (10, 22), since some of the factors that affect digestion patterns of protein are age-related, such as gastric emptying rate (25, 26) and gastric acid secretions (27). In this study, sow parity was highly concentrated at parity 4 and parity 5, and it has to be realized that the effects of protein kinetics may vary in younger parity sows.

Levels of sow blood urea, NEFA, and CREA were evaluated, as they are associated with various metabolic activities and body reserve mobilization. Urea is a common indicator of amino acid catabolism. When amino acids are not used for protein synthesis, the carbon skeletons (α-keto acids) from amino acids will be used as fuel in the liver and oxidized, the remaining amine part of amino acids, in the form of ammonia, will participate in the urea cycle with carbamoyl phosphate synthetase-1, and urea will be released to the blood before being excreted *via* the kidney (28). Urea can therefore be of dietary origin, however, may also originate from mobilized protein reserves. In this study, a high fast protein intake led to higher plasma urea level throughout the lactation, which indicates that fast released amino acids were more readily catabolized to be used as energy, rather than being used for protein synthesis, which matches the higher level of estimated body protein losses compared to sows fed slow protein. This is in line with the results which indicated that the fast protein intake (whey protein) in humans resulted in a higher percentage of dietary nitrogen to be lost as urea, compared to the slow protein (casein) intake (28 > 22%, *p* < 0.05) (11).

Creatinine in the circulation is recognized as an indicator of protein mobilization. As expected, sow plasma CREA was positively related to estimated protein reserve loss in this study (β = 0.004 μmol/L⋅kg^–1^, *p* = 0.02). However, even though sows fed with a high level of slow protein had the lowest estimated protein loss and a tendency for lower loin muscle loss, plasma CREA in these sows was only numerically lower during lactation. Similarly, increasing levels of dietary protein (from 12.8 to 17.8%) during lactation in multiparous sows tended to reduce estimated body protein loss but did not affect blood CREA levels (29). Nevertheless, multiparous sows fed 20.1% compared to 18.8% crude protein had lower blood CREA concentration at weaning (1.65 vs. 1.85 mg/dL, *p* < 0.05), which was accompanied by a lower body weight loss (20.1 vs. 23.4 kg) (7). One of the reasons that no differences were found in plasma CREA between treatments in this study could be that blood samples were collected 4 h after the morning feeding, at a time when protein mobilization may be temporarily suppressed by dietary protein intake.

Non-esterified fatty acid, an intermediate product from triglycerides to acetyl CoA for supporting ATP synthesis, is widely recognized as a marker for adipose tissue catabolism (30–32). In these results, plasma NEFA appeared to correlate positively with estimated fat loss and backfat loss (β = 4.68 μmol/L kg^–1^, *p* < 0.01; β = 2.71 μmol/L mm^–1^, *p* < 0.01, respectively). Given that there were no differences between treatments in fat loss and backfat loss, we expected to see no differences in NEFA, whereas on Day 6, sows fed a diet high in slow protein tended to have higher plasma NEFA than sows fed a diet low in slow protein. We speculate that the energy requirement of milk protein synthesis in sows fed more slow protein was compensated by fat tissue mobilization, due to a lack of catabolic use of amino acid. The protein turnover for milk synthesis is an energetically costly process (33). An increasing concentration of dietary protein increased backfat mobilization, which suggested that more energy was required with an increased level of available amino acids (8). In our study, in sows fed with more fast protein, a higher proportion of the energy used for protein synthesis might have come from local catabolic use of amino acids, as urea levels were higher in these sows. Furthermore, fast protein intake was found to negatively correlate with plasma NEFA level (*r* = −0.37, *p* < 0.01), and slow protein intake was not related to NEFA level. This suggests that fast protein can better cover the energy demand during the absorption and synthesis of NEFA with stronger catabolic use of amino acid than slow protein.

Insulin-like growth factor-1 is also considered as a key metabolic indicator in lactating sows. A low IGF-1 level is commonly associated with a greater negative energy balance (34). In this study, plasma IGF-1 did not show significant relationships with body weight, protein, or fat reserve loss, and moreover, IGF-1 did not respond to the slow protein level in the sow diet. Similarly, sows fed with either 10.5 or 16.6% dietary crude protein, lost 17.3 and 10.6% of their body protein during lactation (*p* < 0.001), had similar blood IGF-1 levels at weaning (54.9 vs. 57.4, ng/mL, *p* = 0.322) (35). However, sows that lost more protein mass (4.1 kg) during lactation were found to have lower plasma IGF-1 level before weaning than sows that lost less protein mass (2.0 kg; *p* < 0.01) (36). Across studies, sows that had lower plasma IGF-1 level had higher total weight loss at lactation than those sows without differences in IGF-1 level (19–30 > 10–17 kg) (35, 36). This suggests that IGF-1 level may only respond when sows have higher degree of body reserve mobilization.

The metabolic status of sows in lactation is crucial to the growth of suckling piglets when it impacts on milk production. In this study, different levels of slow protein in the sow diet did not elicit changes in milk protein, fat, or lactose concentrations. Also, as a reflection of total milk production, the total weight gain of litters did not respond to the level of dietary slow protein. In lactating sows, milk production is recognized to have highest priority (37), and given that the protein derived from body protein tissue mobilization can only account for a small part of milk protein (6), the milk protein may largely originate from dietary protein. The previous studies indicated that elevating dietary protein level could increase milk protein output (8, 38, 39) and improved weight gain of suckling litters (8, 40). When dietary protein supply is insufficient, sows will mobilize more body protein tissue to support milk production (6). Therefore, for this study, it was expected that slow protein could be a better source for milk synthesis, given that sows fed with higher level of slow protein maintained the same ability to sustain milk production while mobilizing less body protein. However, it was not measured to what extent milk protein content originated from dietary protein or from mobilized maternal protein reserves. To confirm the beneficial effect of slow dietary protein on the use of protein for milk, further studies powered by isotopic tracing technique are needed. Furthermore, as feeding fast protein repeatedly could slow down its digestion rate (10), the contrast between fast and protein sources might be reduced in this study, as diets were given to sows in three repeated meals per day, and therefore, the potential effects of protein kinetics on total milk production might be attenuated.

For modern pig production, feeding sows in a more environmentally friendly way is of significant importance to achieve sustainability. Thus, this study investigated whether protein kinetics can improve protein utilization and thereby reduce nitrogen excretion. Total nitrogen output was not different between the treatments. However, sows fed with a high level of slow protein had more nitrogen in their feces than sows fed with low or medium levels of slow protein, because of the lower apparent digestibility of the slow protein diet compared to the fast protein diet. By contrast, the nitrogen output in urine was estimated to be higher in sows fed with low slow protein than sows subjected to high slow protein, because of the increased deamination of amino acids. This was supported by increased conversion of ingested nitrogen to urea after fast protein (soy protein) uptake than slow protein (milk protein) uptake by humans (11).

## Conclusion

Feeding protein sources with a high fraction of slowly degradable protein to multiparous sows reduced body weight loss and protein mobilization during lactation, without affecting litter growth performance. The high level of slow protein likely reduced oxidation of amino acids, as evidenced by lower blood plasma urea level throughout lactation, thereby increasing the available substrate for milk protein synthesis. The exact metabolism of nitrogen and the use of dietary and maternal protein for milk production and final losses to urine and faces need to be established in further studies.

## Data Availability Statement

The raw data supporting the conclusions of this article will be made available by the authors, without undue reservation.

## Ethics Statement

The animal study was reviewed and approved by China Agricultural University Animal Care and Use Committee.

## Author Contributions

HY: conceptualization, software, formal analysis, investigation, and writing – original draft. PL: conceptualization, methodology, software, formal analysis, writing – review and editing, and supervision. NJ: methodology and writing – review and editing. YW, YB, DL, GP, and DH: conceptualization and writing – review and editing. BK: conceptualization, writing - review and editing, and supervision. NS: conceptualization, methodology, formal analysis, writing – review and editing, and supervision. JW: conceptualization, methodology, resources, writing – review and editing, project administration, funding acquisition, and supervision. All authors contributed to the article and approved the submitted version.

## Conflict of Interest

PL, NJ, and GP are employed by Trouw Nutrition R&D. The remaining authors declare that the research was conducted in the absence of any commercial or financial relationships that could be construed as a potential conflict of interest.

## Publisher’s Note

All claims expressed in this article are solely those of the authors and do not necessarily represent those of their affiliated organizations, or those of the publisher, the editors and the reviewers. Any product that may be evaluated in this article, or claim that may be made by its manufacturer, is not guaranteed or endorsed by the publisher.

## References

[1] Tokach M Menegat M Gourley K Goodband R. Nutrient requirements of the modern high-producing lactating sow, with an emphasis on amino acid requirements. *Animal.* (2019) 13:2967–77. 10.1017/S1751731119001253 31199216

[2] Yagüe AP. Use of hyperprolific sows and implications. In: González R editor. *Nutition of Hyperprolific Sows*. Madrid: E Novus International, Inc. (2019). p. 11–38.

[3] Quesnel H Mejia-Guadarrama CA Dourmad J-Y Farmer C Prunier A. Dietary protein restriction during lactation in primiparous sows with different live weights at farrowing: I. consequences on sow metabolic status and litter growth. *Reprod Nutr Dev.* (2005) 45:39–56. 10.1051/rnd:2005004 15865055

[4] Schenkel A Bernardi M Bortolozzo F Wentz I. Body reserve mobilization during lactation in first parity sows and its effect on second litter size. *Livest Sci.* (2010) 132:165–72.

[5] Costermans NG Teerds KJ Middelkoop A Roelen BA Schoevers EJ van Tol HT Consequences of negative energy balance on follicular development and oocyte quality in primiparous sows. *Biol Reprod.* (2020) 102:388–98. 10.1093/biolre/ioz175 31504218 PMC7016286

[6] Costermans NG Soede NM Middelkoop A Laurenssen BF Koopmanschap RE Zak L Influence of the metabolic state during lactation on milk production in modern sows. *Animal.* (2020) 14:2543–53. 10.1017/S1751731120001536 32580816

[7] Yang Y Heo S Jin Z Yun J Choi J Yoon S Effects of lysine intake during late gestation and lactation on blood metabolites, hormones, milk composition and reproductive performance in primiparous and multiparous sows. *Anim Reprod Sci.* (2009) 112:199–214. 10.1016/j.anireprosci.2008.04.031 18547756

[8] Strathe AV Bruun TS Geertsen N Zerrahn J-E Hansen CF. Increased dietary protein levels during lactation improved sow and litter performance. *Anim Feed Sci Technol.* (2017) 232:169–81.

[9] Oenema O. Governmental policies and measures regulating nitrogen and phosphorus from animal manure in European agriculture. *J Anim Sci.* (2004) 82(Suppl. 13):E196–206. 10.2527/2004.8213_supplE196x 15471798

[10] Dangin M Guillet C Garcia-Rodenas C Gachon P Bouteloup-Demange C Reiffers-Magnani K The rate of protein digestion affects protein gain differently during aging in humans. *J Physiol.* (2003) 549(Pt 2):635–44. 10.1113/jphysiol.2002.036897 12665610 PMC2342962

[11] Fouillet H Juillet B Gaudichon C Mariotti F Tome D Bos C. Absorption kinetics are a key factor regulating postprandial protein metabolism in response to qualitative and quantitative variations in protein intake. *Am J Physiol Regul Integr Comp Physiol.* (2009) 297:R1691–705. 10.1152/ajpregu.00281.2009 19812354

[12] Boirie Y Dangin M Gachon P Vasson M-P Maubois J-L Beaufrère B. Slow and fast dietary proteins differently modulate postprandial protein accretion. *Proc Natl Acad Sci USA.* (1997) 94:14930–5. 10.1073/pnas.94.26.14930 9405716 PMC25140

[13] Tang JE Moore DR Kujbida GW Tarnopolsky MA Phillips SM. Ingestion of whey hydrolysate, casein, or soy protein isolate: effects on mixed muscle protein synthesis at rest and following resistance exercise in young men. *J Appl Physiol.* (2009) 107:987–92. 10.1152/japplphysiol.00076.2009 19589961

[14] Chen H Wierenga P Hendriks W Jansman A. In vitro protein digestion kinetics of protein sources for pigs. *Animal.* (2019) 13:1154–64. 10.1017/S1751731118002811 30370898

[15] National Research Council. *Nutrient Requirements of Swine*. 11th ed. Washington, DC: National Academies Press (2012).

[16] Association of Official Analytical Chemists [AOAC]. *Official Methods of Analysis*. 18th ed. Gaithersburg, MD: AOAC International (2007).

[17] Atkinson J Hilton J Slinger S. Evaluation of acid-insoluble ash as an indicator of feed digestibility in rainbow trout (*Salmo gairdneri*). *Can J Fish Aquat Sci.* (1984) 41:1384–6.

[18] Noblet J Henry Y. Energy evaluation systems for pig diets: a review. *Livest Prod Sci.* (1993) 36:121–41.

[19] Dourmad J Etienne M Noblet J Causeur D. Prédiction de la composition chimique des truies reproductrices à partir du poids vif et de l’épaisseur de lard. *J Recherche Porcine France.* (1997) 29:255–62.

[20] Noblet J Etienne M. Estimation of sow milk nutrient output. *J Anim Sci.* (1989) 67:3352–9. 10.2527/jas1989.67123352x 2613581

[21] Dourmad J-Y Etienne M Valancogne A Dubois S van Milgen J Noblet J. Inraporc: a model and decision support tool for the nutrition of sows. *Anim Feed Sci Technol.* (2008) 143:372–86.

[22] Dangin M Boirie Y Garcia-Rodenas C Gachon P Fauquant J Callier P The digestion rate of protein is an independent regulating factor of postprandial protein retention. *Am J Physiol Endocrinol Metab.* (2001) 280:E340–8. 10.1152/ajpendo.2001.280.2.E340 11158939

[23] Fouillet H Mariotti F Gaudichon C Bos C Tomeì D. Peripheral and splanchnic metabolism of dietary nitrogen are differently affected by the protein source in humans as assessed by compartmental modeling. *J Nutr.* (2002) 132:125–33. 10.1093/jn/132.1.125 11773519

[24] Earl D Hindley ST. The rate-limiting step of protein synthesis in vivo and in vitro and the distribution of growing peptides between the puromycinlabile and puromycin-non-labile sites on polyribosomes. *Biochem J.* (1971) 122:267–76. 10.1042/bj1220267 5118100 PMC1176775

[25] Clarkston W Pantano M Morley J Horowitz M Littlefield J Burton F. Evidence for the anorexia of aging: gastrointestinal transit and hunger in healthy elderly vs. young adults. *Am J Physiol Regul Integr Comp Physiol.* (1997) 272:R243–8. 10.1152/ajpregu.1997.272.1.R243 9039015

[26] Cook CG Andrews JM Jones KL Wittert GA Chapman IM Morley JE Effects of small intestinal nutrient infusion on appetite and pyloric motility are modified by age. *Am J Physiol Regul Integr Comp Physiol.* (1997) 273:R755–61. 10.1152/ajpregu.1997.273.2.R755 9277565

[27] Achour L Meance S Briend A. Comparison of gastric emptying of a solid and a liquid nutritional rehabilitation food. *Eur J Clin Nutr.* (2001) 55:769–72. 10.1038/sj.ejcn.1601221 11528491

[28] Brady S. *Basic Neurochemistry: Molecular, Cellular and Medical Aspects.* Amsterdam: Elsevier (2005).

[29] Strathe A Bruun T Tauson A-H Theil P Hansen C. Increased dietary protein for lactating sows affects body composition. Blood metabolites and milk production. *Animal.* (2020) 14:285–94. 10.1017/S1751731119001678 31368423

[30] Xue L Piao X Li D Li P Zhang R Kim SW The effect of the ratio of standardized ileal digestible lysine to metabolizable energy on growth performance, blood metabolites and hormones of lactating sows. *J Anim Sci Biotechnol.* (2012) 3:1–12. 10.1186/2049-1891-3-11 22958422 PMC3436621

[31] Hojgaard CK Bruun TS Strathe AV Zerrahn J-E Hansen CF. High-yielding lactating sows maintained a high litter growth when fed reduced crude protein, crystalline amino acid-supplemented diets. *Livest Sci.* (2019) 226:40–7. 10.1016/j.livsci.2019.06.002

[32] Weissensteiner R Baldinger L Hagmüller W Zollitsch W. Effects of two 100% organic diets differing in proportion of home-grown components and protein concentration on performance of lactating sows. *Livest Sci.* (2018) 214:211–8. 10.1016/j.livsci.2018.06.006

[33] Bionaz M Hurley W Loor J. Milk protein synthesis in the lactating mammary gland: insights from transcriptomics analyses. *Milk Protein.* (2012) 11:285–324.

[34] Mao J Zak L Cosgrove J Shostak S Foxcroft G. Reproductive, metabolic, and endocrine responses to feed restriction and Gnrh treatment in primiparous, lactating sows. *J Anim Sci.* (1999) 77:724–35. 10.2527/1999.773724x 10229370

[35] Clowes E Aherne F Schaefer A Foxcroft G Baracos V. Parturition body size and body protein loss during lactation influence performance during lactation and ovarian function at weaning in first-parity sows. *J Anim Sci.* (2003) 81:1517–28. 10.2527/2003.8161517x 12817500

[36] Mejia-Guadarrama C Pasquier A Dourmad J-Y Prunier A Quesnel H. Protein (lysine) restriction in primiparous lactating sows: effects on metabolic state, somatotropic axis, and reproductive performance after weaning. *J Anim Sci.* (2002) 80:3286–300. 10.2527/2002.80123286x 12542170

[37] Strathe A Bruun T Hansen C. Sows with high milk production had both a high feed intake and high body mobilization. *Animal.* (2017) 11:1913–21. 10.1017/S1751731117000155 28196552

[38] Guan X Pettigrew J Ku P Ames N Bequette B Trottier N. Dietary protein concentration affects plasma arteriovenous difference of amino acids across the porcine mammary gland. *J Anim Sci.* (2004) 82:2953–63. 10.2527/2004.82102953x 15484947

[39] Laspiur JP Burton JL Weber PS Moore J Kirkwood RN Trottier NL. Dietary protein intake and stage of lactation differentially modulate amino acid transporter mrna abundance in porcine mammary tissue. *J Nutr.* (2009) 139:1677–84. 10.3945/jn.108.103549 19625700

[40] Yang H Pettigrew J Johnston LJ Shurson GC Wheaton J White ME Effects of dietary lysine intake during lactation on blood metabolites, hormones, and reproductive performance in primiparous sows. *J Anim Sci.* (2000) 78:1001–9. 10.2527/2000.7841001x 10784191

